# Inorganic Chemical Fertilizer Application to Wheat Reduces the Abundance of Putative Plant Growth-Promoting Rhizobacteria

**DOI:** 10.3389/fmicb.2021.642587

**Published:** 2021-03-11

**Authors:** Tessa E. Reid, Vanessa N. Kavamura, Maïder Abadie, Adriana Torres-Ballesteros, Mark Pawlett, Ian M. Clark, Jim Harris, Tim H. Mauchline

**Affiliations:** ^1^Sustainable Agriculture Sciences, Rothamsted Research, Harpenden, United Kingdom; ^2^Cranfield Soil and Agrifood Institute, Cranfield University, Cranfield, United Kingdom

**Keywords:** rhizosphere, rhizoplane, fertilizer, microbiome, bacteria, plant growth-promoting rhizobacteria

## Abstract

The profound negative effect of inorganic chemical fertilizer application on rhizobacterial diversity has been well documented using 16S rRNA gene amplicon sequencing and predictive metagenomics. We aimed to measure the function and relative abundance of readily culturable putative plant growth-promoting rhizobacterial (PGPR) isolates from wheat root soil samples under contrasting inorganic fertilization regimes. We hypothesized that putative PGPR abundance will be reduced in fertilized relative to unfertilized samples. *Triticum aestivum* cv. Cadenza seeds were sown in a nutrient depleted agricultural soil in pots treated with and without Osmocote^®^ fertilizer containing nitrogen-phosphorous-potassium (NPK). Rhizosphere and rhizoplane samples were collected at flowering stage (10 weeks) and analyzed by culture-independent (CI) amplicon sequence variant (ASV) analysis of rhizobacterial DNA as well as culture-dependent (CD) techniques. Rhizosphere and rhizoplane derived microbiota culture collections were tested for plant growth-promoting traits using functional bioassays. In general, fertilizer addition decreased the proportion of nutrient-solubilizing bacteria (nitrate, phosphate, potassium, iron, and zinc) isolated from rhizocompartments in wheat whereas salt tolerant bacteria were not affected. A “PGPR” database was created from isolate 16S rRNA gene sequences against which total amplified 16S rRNA soil DNA was searched, identifying 1.52% of total community ASVs as culturable PGPR isolates. Bioassays identified a higher proportion of PGPR in non-fertilized samples [rhizosphere (49%) and rhizoplane (91%)] compared to fertilized samples [rhizosphere (21%) and rhizoplane (19%)] which constituted approximately 1.95 and 1.25% in non-fertilized and fertilized total community DNA, respectively. The analyses of 16S rRNA genes and deduced functional profiles provide an in-depth understanding of the responses of bacterial communities to fertilizer; our study suggests that rhizobacteria that potentially benefit plants by mobilizing insoluble nutrients in soil are reduced by chemical fertilizer addition. This knowledge will benefit the development of more targeted biofertilization strategies.

## Introduction

Since anthropogenic plant domestication began ca. 19,000 years ago, edible plants, e.g., cereals have been extensively bred (Lev-Yadun et al., 2000; Tanno and Willcox, 2006). However, current high-yielding dwarf crop varieties rely on unsustainable levels of inorganic nitrogen and phosphorous fertilizers, pesticides, and other chemical inputs which are environmentally harmful (Rees et al., 2013; Van Grinsven et al., 2013). Wheat is the third most cultivated cereal in the world; the Cambric Arenosol (FAO) predicts that by 2050 the global population will reach 9.73 billion meaning food production must be accordingly increased by 50% (FAO, 2017). By 2027, demand for wheat will increase to 833 million tons, which is 10% above the annual current production (OECD/FAO, 2018).

Soil microbial communities influence plant growth, health, and resource use efficiency, especially the subset that coexist and are selected to form the root microbiome (Berendson et al., 2012; Mendes et al., 2013; Schlaeppi and Bulgarelli, 2015; Mauchline and Malone, 2017). A gradient of intimacy between plant roots and microbes extends away from the root: plant influence over the microbial community increases nearer the root surface. Root surface microbes are said to inhabit the rhizoplane, and those in soil closely associated with the root, the rhizosphere (Hiltner, 1904; Zhang et al., 2017). Rhizosphere and rhizoplane microorganisms can benefit crop plants in several ways including improved plant nutritional status and protection against biotic and abiotic stresses (Bloemberg and Lugtenberg, 2001; Turner et al., 2013; Choudhary et al., 2016; Ahkami et al., 2017), so are termed plant growth-promoting rhizobacteria (PGPR) (Kloepper and Schroth, 1978). Inoculating plants with PGPR can stimulate crop growth, forming the basis for the biofertilizer industry providing green alternatives to synthetic fertilizers and agrochemicals (Backer et al., 2018). Understanding the effect of agricultural practices, such as fertilization regime, on PGPR populations is essential to optimize microbiome function in the sustainable intensification of agriculture (Hartmann et al., 2015).

To date, microbial community studies have focused on taxonomic composition, but the functional potential of the microbiome may be more important to ensure key functions for holobiont fitness (Bai et al., 2015; Lemanceau et al., 2017). With advances in next-generation sequencing technologies, the wheat microbiome has mostly been defined based on culture-independent (CI) methods; host genotype (Mahoney et al., 2017), fertilization regime (Kavamura et al., 2018; Chen et al., 2019), land management and seed load (Kavamura et al., 2019), irrigation (Mavrodi et al., 2018), growth stage (Chen et al., 2019), and dwarfing (Kavamura et al., 2020) all affect the rhizosphere community structure. However, little has been done to link taxonomic structure of the wheat microbiome to its functional ability.

Using CI and culture-dependent (CD) methods, we studied the effect of chemical fertilizer on putative PGPR abundance in the commercial wheat variety, Cadenza, from a low input agricultural soil depleted in most nutrients, in which beneficial microorganisms are important to sustain crop production. We hypothesized that the abundance of rhizobacteria with plant growth-promoting traits would be reduced for fertilized relative to unfertilized wheat due to differences in plant nutrient status. Addition of nitrogen-phosphorous-potassium (NPK) would mean that plants no longer need to interact with beneficial rhizobacteria to provide nutrients to sustain growth. Our aim was to characterize culturable bacteria with plant growth-promoting traits; determine their abundance within culturable communities; and characterize CI and CD 16S rRNA gene DNA to assess the impact of the widely used NPK fertilizer on putative PGPR populations. This was achieved by creating isolate libraries from each soil sample and subjecting them to a range of bioassays which test key traits in nutrient acquisition to establish the abundance of isolates with beneficial traits. 16S rRNA gene sequences from isolates were used to create a “PGPR” database against which total CI amplified 16S rRNA soil DNA was searched in order to determine the relative abundance of culturable PGPR within total community DNA.

## Materials and Methods

### Soil Collection, Experimental Setup, and Harvesting

We evaluated the rhizosphere and rhizoplane soil from wheat grown with and without NPK fertilizer. Soil was collected from Stackyard bare-fallow soil mine (LATLONG 52.000293N, -0.614308E), a well-draining sandy loam soil from the Rothamsted Research experimental farm at Woburn, Bedfordshire (United Kingdom). The soil is a Cottenham series (Catt et al., 1980) classified as a FAO, chosen to reduce the legacy effect of prior cropping systems. Soil was sieved (2 mm mesh), mixed thoroughly, and stored at 4°C in polythene bags prior to use. *Triticum aestivum* cv. Cadenza seeds were surface sterilized [70% ethanol, 10 min; 1.5% active chlorine, 1 h; 5 × rinse, sterile distilled water (SDW); overnight imbibition, sterile water, 4°C] before germination on filter paper. Seedlings were planted (1× seedling/pot; 9 × 9 × 10 cm pots, ∼500 g soil) with and without NPK granules [15% N, 9% P_2_O_5_, 11% K_2_O, 2% MgO with micro-nutrients (B, Cu, Fe, Mn, Mo, and Zn); Osmocote, United Kingdom] (∼2.5 g per pot). Four replicate pots were prepared for each treatment. Plants were grown in a glasshouse (20°C, 16 h/day light regime) and watered daily with tap water.

Pots were harvested at the start of flowering (Zadoks growth stage 61; 10 weeks post germination) (Zadoks et al., 1974), resulting in eight rhizosphere samples and eight root samples. Height (from soil surface to head of longest stem) was measured, then soil was gently tipped from the pot onto a fresh polythene bag. Loose soil was discarded and non-rhizospheric soil carefully removed. Roots were vigorously shaken in a bag to release tightly attached soil (i.e., rhizosphere) and mixed to homogenize. The root system was excised, cut vertically in half and placed in sterile 10 ml vials for subsequent rhizoplane work. One half was frozen (−20°C) for soil DNA extraction and the other stored (4°C) for culture work. Around 5 g of rhizosphere soil and 1 g of root was collected per plant. The remainder of the plant was dried (80°C, 24 h) and dry foliar plant biomass measured.

### Isolation of Bacteria

To obtain a library of rhizospheric bacteria, 1 g of each rhizosphere soil sample was diluted 10-fold (SDW) and shaken vigorously for 10 min using a shaker. To increase the diversity and number of culturable isolates returned in this study, rhizosphere samples were plated onto both 10TSA and an additional seven agar types (Supplementary Table 1; Bai et al., 2015). Soil suspensions were serially diluted and 100 μl of final dilution spread on agar (six replicates per agar type). Different dilutions were plated depending on agar type (Supplementary Table 2) and incubated (25°C) for 4 days. Individual colonies were picked and inoculated in 500 μl tryptone soya broth (TSB) (Oxoid, Basingstoke, United Kingdom) (1/10th concentration) in deep well 96-well plates (Supplementary Figure 1) and incubated (25°C, 2 days). For rhizoplane-colonizing bacteria, root samples were weighed and 900 μl SDW was added for every 0.1 g root. Samples were shaken vigorously for 10 min using a shaker, serially diluted, plated onto 10TSA and incubated (25°C, 4–6 days). Individual colonies were picked and inoculated in 500 μl TSB (1/10th concentration) (96-well plates, 25°C, 2 days) prior to functional analysis.

In addition, once colonies had been hand-picked from agar plates the remaining microbial biomass was resuspended in 5 ml SDW using a sterile spreader and transferred to a 50 ml centrifuge tube. Suspensions from technical replicates of the same agar (Supplementary Figure 2) were combined and each sample vortexed (10 min, high speed) to ensure homogeneity. Sub-samples of each were stored (2 ml Eppendorf tube, −20°C) for genomic DNA extraction.

### Bioassays for Plant Growth-Promoting Traits

#### Bioassay Inoculation

Plant growth-promoting functions were tested using previously established bioassays. A sterile 48-prong inoculating manifold was used to spot individual inoculated isolates from the 96-well plate liquid cultures onto agar (2× technical replicates per 96-well plate). Assays were incubated (25°C, 5–7 days); positive isolates were counted per sample for each functional assay.

#### Hydrolyzation of Casein

Casein agar is used to detect peptide bond hydrolyzing microorganisms (Frazier and Rupp, 1928). Isolate cultures were spot inoculated onto agar supplemented with skimmed milk powder as the casein source. Casein agar (Hardy Diagnostics, Santa Maria, CA, United States): 5% skimmed milk powder, 0.5% pancreatic digest of casein, 0.25% yeast extract, 0.1% D-glucose, 1.25% agar. Hydrolyzing isolates produced a clear halo in the surrounding medium.

#### Solubilization of Insoluble Phosphate, Potassium, and Zinc

Isolate cultures were spot inoculated onto agar plates containing: tricalcium phosphate as an insoluble phosphate source (Pikovskaya, 1948); potash feldspar (Bath potters, United Kingdom) as an insoluble potassium source (Zhang and Kong, 2014); and zinc oxide as an insoluble zinc source (HiMedia M2023) (Subba Rao, 1977). Pikovskayas agar (Pikovskaya, 1948): 0.05% yeast extract, 1% D-glucose, 0.5% Ca_3_(PO_4_)_2_, 0.05% (NH_4_)_2_SO_4_, 0.02% KCl, 0.01% MgSO_4_•7H_2_O, 0.00001% MnSO_4_•H_2_O, 0.00001% FeSO_4_•7H_2_O, 1.5% agar. Aleksandrov agar (Aleksandrov et al., 1967): 0.05% MgSO_4_•7H_2_O, 0.01% CaCO_3_, 0.2% potash feldspar, 0.5% D-glucose, 0.0005% FeCl_3_•6H_2_O, 0.2% Ca_3_(PO_4_)_2_, 2% agar, pH 7.0–7.2. Zinc solubilizing agar (Subba Rao, 1977): 1% D-glucose, 0.1% (NH_4_)_2_SO_4_, 0.02% KCl, 0.01% K_2_HPO_4_, 0.02% MgSO_4_•7H_2_O, 0.1% ZnO, 1.5% agar. The plates were incubated at 25°C for 5–7 days and observed for the formation of halo zone around the colonies.

#### Production of Siderophores

Iron solubilization was tested using agar containing chrome azurol S (CAS) and hexadecyltrimethylammonium bromide (HDTMA) which form a blue color complex with ferric iron; a color change to orange is observed when a strong iron chelator such as a siderophore removes iron from the dye complex (Schwyn and Neilands, 1987; Louden et al., 2011). The medium was prepared as outlined in Louden et al. (2011). Iron solubilization was denoted as either “positive” or “negative” based on the presence, or absence of an orange halo surrounding the colony.

#### Salt Tolerance

*In vitro* screening of the isolates tolerance to salt stress was tested by culturing strains on 10TSA supplemented with 5% (w/v) sodium chloride (NaCl). 10TSA with no additional NaCl was included as a control.

#### Statistical Analyses for Culture-Dependent Work

Statistical differences in the frequency of positive vs. negative isolates (*n* = 376) between non-fertilized and fertilized wheat were performed in R 3.6.1^1^ using the “chisq.test” function. Box plots were created in GraphPad Prism version 8 for Mac (GraphPad Software, Inc., San Diego, CA, United States). This software was also used to calculate two-way analysis of variance (ANOVA) and pair-wise *t*-tests with Šidák correction for bacterial abundance and absolute abundance of nutrient-solubilizing isolates. Data were first normalized by logarithmic transformation. Normality was confirmed by quantile–quantile normality plots and Shapiro–Wilk test; homogeneity of variances was confirmed by residuals vs. fits plots and Spearman’s test for heteroscedasticity.

### DNA Analysis

#### Mixed Culture DNA Extraction and Quantitation

Each mixed culture sample was subjected to Sigma GenElute Bacterial Genomic DNA extraction kit using the lysozyme utilizing Gram-positive bacterial preparation method to ensure lysis of both Gram-positive and Gram-negative cells, according to the manufacturer’s instructions. DNA purity and concentrations were established by NanoDrop spectrophotometry (Thermo Fisher Scientific, Wilmington, DE, United States), and a Qubit 2.0 Fluorimeter using the dsDNA HS assay kit (Thermo Fisher Scientific), respectively.

#### Soil DNA Extraction and Quantitation

For each sample, total soil DNA was extracted from approximately 0.25 g homogenized soil [Qiagen DNeasy PowerSoil DNA isolation kit (Venlo, Netherlands)], according to the manufacturer’s instructions using the MP Biomedicals FastPrep-24 machine twice (30 s, 5.5 m s^–1^). DNA concentrations and purity were determined as above.

#### Amplicon Library Preparation and Sequencing

To assess the impact of fertilizer on microbial community composition, bacterial 16S rRNA gene amplicons were subjected to Illumina^®^ sequencing using the MiSeq platform. Amplicons (∼460 bp) spanning the V3-V4 hypervariable region of the 16S rRNA gene were produced using primers 341F (5′-CCTACGGGAGGCAGCAG-3′) and 806R (5′-GGACTACHVGGGTWTCTAAT-3′) (Klindworth et al., 2013). Rhizosphere DNA was sent to Novogene (HK) (Wan Chai, Hong Kong) for 2 × 250 bp paired-end sequencing on a MiSeq instrument. Rhizoplane and bacterial samples were sequenced in-house (2 × 300 bp paired-end sequencing) on a MiSeq instrument; see Supplementary Methods for full details. Amplicon preparation followed the protocol of Kozich et al. (2013).

#### Processing of 16S rRNA Gene Amplicon Sequence Data

Demultiplexing of raw sequences was performed by CASAVA data analysis software (Illumina). Paired-end sequences were merged using the vsearch merge_pairs function (Rognes et al., 2016) then filtered, de-replicated, and denoised to identify amplicon sequence variants (ASVs) using the DADA2 1.2 (Callahan et al., 2016) pipeline with Quantitative Insights into Microbial Ecology (QIIME2) (version 2018.11.0) default parameters (Bolyen et al., 2019). The resulting ASV table retained high quality non-chimeric reads and was used to build a phylogenetic tree using the align-to-tree-mafft-fasttree command in QIIME2. Taxonomy was assigned using the SILVA132 database (Quast et al., 2012; Yilmaz et al., 2013). All non-bacterial ASVs were removed for further analysis.

#### Colony PCR for Identification of Rhizobacterial Isolates

To identify individual rhizobacterial isolates, amplicons (∼1,500 bp) spanning almost the full length of the 16S rRNA gene were produced using primers fD1 (5′-AGAGTTTGATCCTGGCTCAG-3′) and rD1 (5′-AAGGAGGTGATCCAGCC-3′). From a total of 1,504 rhizobacterial isolates (94 isolates per rhizosphere sample; 94 isolates per rhizoplane sample), 541 isolates were sequenced to gain a representative population of species present in non-fertilized and fertilized wheat. Amplicons were produced by colony PCR and sent to Eurofins Genomics Germany for purification and Sanger sequencing; see Supplementary Methods for full details. Amplicon sequences were processed in Geneious Prime version 2020.1.1; taxonomically assigned using the SILVA Alignment, Classification and Tree (ACT) service^2^; and deposited in NCBI GenBank (for individual accession numbers see Supplementary Data).

### Isolate Database Creation

The resulting 541 16S rRNA gene sequences and corresponding taxonomy were used to create QIIME2 taxonomy and ASV files to identify whether isolates matched major ASVs within the 16S rRNA gene amplicon dataset. The classify-consensus-blast command in QIIME2 was used at 100% sequence identity to search the 16S rRNA gene amplicon sequences against the isolate database. Secondly, a “PGPR” database was created which only included isolate sequences identified as “putative PGPR” (as they were found to be positive for at least one functional trait) to identify the relative abundance of potentially plant beneficial isolates within CI and CD amplicon datasets. ASVs identified as “Bacteria” were considered rhizobacterial isolates.

### Data Visualization and Statistical Analyses

#### Total 16S rRNA Gene Dataset

The resulting ASV table was analyzed in R 3.6.1 using Phyloseq (v1.30.0) (available at https://joey711.github.io/phyloseq/) (McMurdie and Holmes, 2013). Amplicon sequencing data were normalized using DESeq2 to account for differences in sequencing bias (Love et al., 2014), except for alpha diversity analysis, which was calculated by normalizing sequence number to minimum sample size (8,554) by random subsampling. The subsampling of sequences still yielded sufficient resolution of bacterial communities, as suggested by rarefaction curve analysis (Supplementary Figure 3). Two-way type III ANOVA was performed using the R function “aov” with fertilizer and rhizocompartment as factors, to determine the dominant factor contributing to variation in means for alpha diversity data. Normality was confirmed by quantile-quantile normality plots and the Shapiro–Wilk test; homogeneity of variances was confirmed by residuals vs. fits plots and the Levene’s test [R package: car (v3.0-6)]. Beta diversity was determined by Principle Coordinate Analysis (PCoA) which was employed on weighted UniFrac distance matrices using the “ordinate” function in the Phyloseq package; significantly different clusters were determined using “adonis” with the “betadisper” test to check for equal variance [R package: vegan (v2.5.6)] (Oksanen et al., 2019). Phylum level community composition was investigated by relative abundance of normalized data, after removal of bacterial ASVs only classified to Kingdom level and ASVs assigned as “environmental samples” for visualization purposes. Unique and shared ASVs were determined in Excel. To identify ASVs preferentially associated with fertilization and vice versa, the differential relative abundances (fold changes) of ASVs between the different groups were determined. Low abundance ASVs with less than 3 counts in <20% of the samples were removed. This was performed individually for each data set with the DeSeq2 package, using the Wald significance test and the Benjamini–Hochberg *p*-value correction. All graphs were rendered in Prism 8.

#### Isolate and PGPR 16S rRNA Gene Dataset

The resulting isolate ASV table and PGPR ASV table were analyzed using Phyloseq (as described above), normalized using DESeq2 and transformed to relative abundances. Heatmaps were created in Excel. ASVs identified as “Bacteria” were considered rhizobacterial isolates or putative PGPR; the remaining ASVs were unassigned. To identify whether isolates matched ASVs preferentially associated with fertilization and vice versa, the differential relative abundances (fold changes) of ASVs between the different groups were determined, as described above. Statistical differences between mean putative PGPR abundance between treatments were assessed using two-way ANOVA and pair-wise *t*-tests with Šidák correction. Normality was confirmed by quantile–quantile normality plots and Shapiro–Wilk test; homogeneity of variances was confirmed by residuals vs. fits plots and Spearman’s test for heteroscedasticity. Box plots were created in Prism 8.

## Results

### Plant Phenotypical Data

Fertilizer addition increased aerial biomass of wheat from 1.27 ± 0.32 to 4.11 ± 0.95 g (Welch’s two-sample *t*-test: *t* = −5.645, d.f. = 3.67, *p* = 0.006) (Supplementary Figure 4). There were no statistical differences in mean height of non-fertilized vs. fertilized wheat plants (*p* > 0.05) (Supplementary Figure 4).

### Rhizobacterial Community Composition in Culture-Independent and -Dependent Communities

**FIGURE 1:**
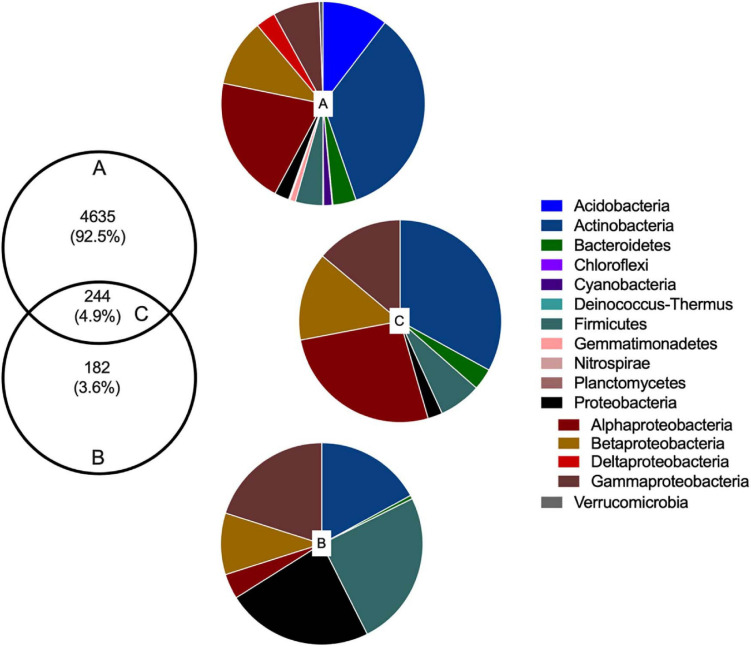
Rhizobacterial taxonomic composition in culture-independent and -dependent communities of wheat grown in soils with and without fertilizer addition. Venn diagram showing the number and proportion of unique ASVs (at 97% similarity) in **(A)** culture-independent and **(B)** culture-dependent communities, and **(C)** shared ASVs detected with both methods. Pie charts correspond to the percentage of bacterial phyla and classes of Proteobacteria assigned to each ASV.

Amplicon-based analysis of the V3–V4 region of the 16S rRNA gene generated a total of 902,187 sequences from 16 CI samples (median 50,658 sequences per sample) and 279,558 sequences from 16 CD samples (median 17,235 sequences per sample). Filtering, denoising, and removal of chimeras resulted in 521,098 (CI) and 206,960 (CD) high-quality sequences, retaining 58 and 70% of initial reads, respectively. The number of sequences per sample ranged from 20,809 to 49,203 (CI) and 12,623 to 24,165 (CD). One CD sample was removed due to low sequence number (<100). ASV analysis using the SILVA132 database generated 4,879 ASVs from CI DNA and 426 ASVs from CD DNA [after removal of ASVs that were not classified as bacteria (archaeal, eukaryote, metagenome, unassigned)]. The CD approach retrieved 4.9% of ASVs from the CI total community; 244 (4.9%) ASVs were found using both CI and CD methods indicating that 182 (3.6%) ASVs detected by the CD method, were absent from the CI DNA dataset (Figure 1).

### Fertilizer Treatment and Niche Effects on Rhizobacterial Community Diversity

**FIGURE 2:**
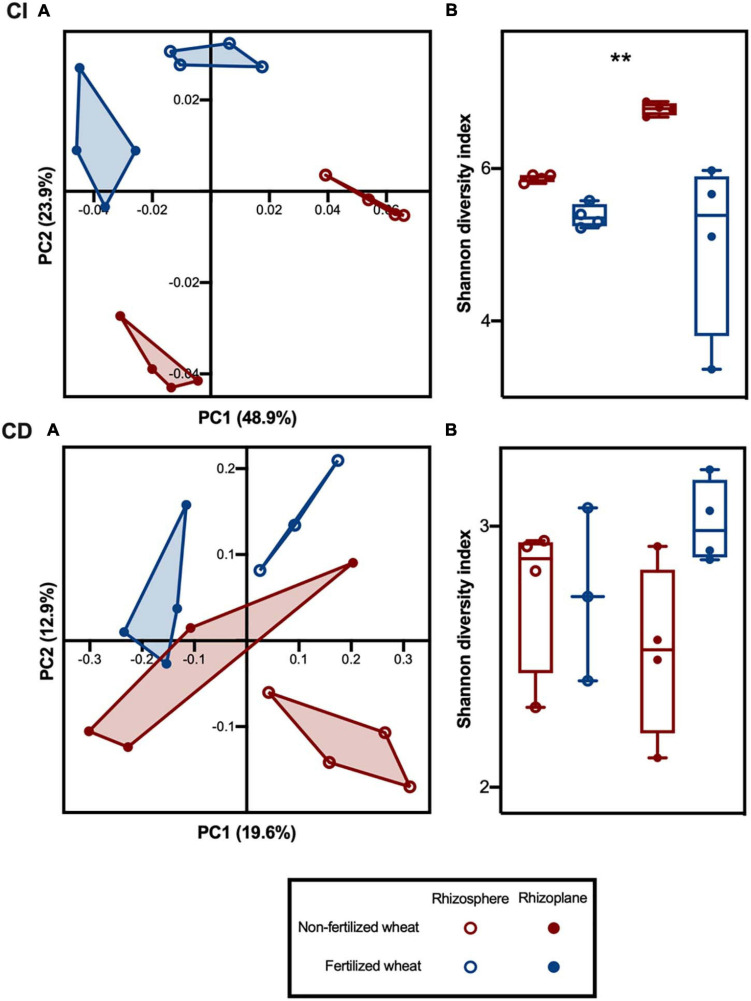
Culture-independent (CI) and culture-dependent (CD) bacterial community diversity in the rhizosphere and rhizoplane of wheat grown in soils with and without fertilizer addition. **(A)** PCoA plots of bacterial composition based on weighted UniFrac distances for CI and CD communities at ASV level. The percentage shown in each axis corresponds to the proportion of variation explained. **(B)** Alpha diversity estimates at ASV level for rhizosphere and rhizoplane bacterial communities in non-fertilized and fertilized wheat; with median (line) and hinges at first and third quartiles (25th and 75th percentiles). Significant differences as determined by two-way type III ANOVA are shown by “**” for *p* < 0.01 between treatment groups. Symbols represent rhizosphere (open circles) and rhizoplane (closed circles) samples from non-fertilized (red) and fertilized (blue) wheat.

Rhizobacterial community beta-diversity, as measured by weighted UniFrac distances, was influenced by both rhizocompartment and fertilizer. PCoA of CI communities showed clustering of samples by rhizocompartment along the first principle coordinate axis (PC1) and clustering by fertilization along the second principle coordinate axis (PC2) (Figure 2A, CI). Two-way permutational analysis of variance (PERMANOVA) indicated that both rhizocompartment and fertilizer effect were significant in CI rhizobacterial communities, as well as their interaction (Supplementary Table 3). Rhizocompartment accounted for 49% of the variance (*p* = 0.001) and fertilizer accounted for 24% of the variance (*p* = 0.001). The two-way PERMANOVA of CD rhizobacterial community beta diversity indicated a significant effect of rhizocompartment (20% of variance, *p* = 0.002), whereas the effect of fertilization was weaker, although still statistically significant (13% of variance, *p* = 0.035), but the interaction was not significant (Supplementary Table 4). PCoA of the CD rhizobacterial community revealed that samples also clustered by rhizocompartment along PC1 (Figure 2A, CD), whereas there was some overlap between treatment groups along PC2, consistent with the small effect size in the PERMANOVA analysis.

CI community alpha diversity, as measured by the Shannon diversity index, was consistent across rhizocompartments but lower in samples from fertilized wheat (Figure 2B, CI). Two-way type III ANOVA indicated that the effect of fertilizer was significant (*p* = 0.0024), while the effects of rhizocompartment and interaction were not (Supplementary Table 5). Rhizobacterial CD communities showed lower alpha diversity compared to CI communities (Figure 2B, CD), with fertilizer and rhizocompartment causing less change in variation (*p* > 0.05) (Supplementary Table 6).

### Comparison of Isolation Media in Assessing the Influence of Fertilizer on Rhizosphere Bacterial Communities

In total, 894 bacterial ASVs were identified from 63 mixed culture samples [four biological replicates from eight agar-types for non-fertilized and fertilized wheat rhizosphere samples (one outlier removed)]. The use of seven additional agar types, as well as 10TSA, retrieved 8.6% of CI ASVs compared to 4.2% from just 10TSA.

**FIGURE 3:**
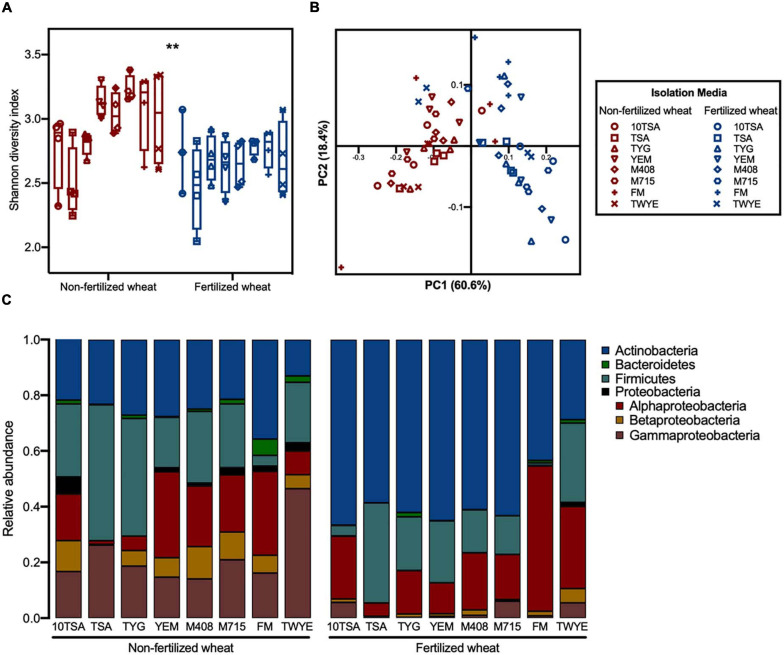
Influence of different culture-agar on bacterial community diversity and structure in the rhizosphere of wheat grown in soils with and without fertilizer. **(A)** Alpha diversity estimates at ASV level for rhizosphere bacterial communities grown on eight agar types with median (line) and hinges at first and third quartiles (25th and 75th percentiles). Significant differences as determined by two-way type III ANOVA are shown by “**” for *p* < 0.01 between treatment groups. **(B)** PCoA plot of bacterial community composition based on weighted UniFrac distances at ASV level, colored by fertilizer treatment. Symbols represent the isolation media that bacteria were cultured on from rhizosphere samples: TSA (10th concentration) (circles), TSA (squares), TYG (up-triangles), YEM (down-triangles), M408 (diamond), M715 (hexagon), FM (plus), TWYE (cross). **(C)** Average relative abundances of bacterial phyla within the bacterial 16S V3–V4 sequences. Identifications are based on the SILVA database for prokaryotes.

Effect of fertilizer was more apparent when a larger number of agar types were used to isolate rhizobacteria. Overall, mean alpha diversity was greater in samples from non-fertilized wheat (2.95 ± 0.31) compared to fertilized wheat (2.66 ± 0.31) (d.f. = 1, *F* = 21, *p* < 0.0001) however, there were no statistical differences between individual means for each agar-type (d.f. = 7, *F* = 3.6, *p* > 0.05) (Figure 3A). Furthermore, fertilizer was the dominant factor contributing to variation in beta diversity (PERMANOVA, *F* = 40, *r*^2^ = 0.35, *p* = 0.001), accounting for 61.7% variation along PC1 compared to 16.7% variation along PC2 as caused by agar-type (PERMANOVA, *F* = 2.6, *r*^2^ = 0.16, *p* = 0.004) (Figure 3B), with no significant interaction. The relative abundance of different taxonomic groups in mixed cultures shifted from non-fertilized wheat plants and fertilized wheat plants (Figure 3C). Most notable, was the higher abundance of Actinobacteria in the rhizosphere of fertilized wheat plants across all media types compared to a higher abundance of Proteobacteria in the rhizosphere of non-fertilized wheat plants.

Agar type had minimal effect on bacterial diversity within treatment groups. There were no statistical differences in mean alpha diversity between different agar types for a given sample from either non-fertilized or fertilized wheat. Furthermore, PCoA showed no distinct groupings between agar type for non-fertilized wheat (Supplementary Figure 5A) (PERMANOVA, *F* = 1.1, *r*^2^ = 0.24, *p* = 0.38) however, fertilized wheat samples from TWYE agar formed a clear grouping compared to the other agar types (Supplementary Figure 5B). With TWYE samples present, media type significantly affected bacterial community structure (PERMANOVA, *F* = 11.9, *r*^2^ = 0.29, *p* = 0.001). However, when TWYE samples were removed from PCoA, agar type had no significant effect on bacterial community structure (PERMANOVA, *F* = 0.86, *r*^2^ = 0.056, *p* = 0.59).

### Effect of Fertilizer on Abundance of Culturable Rhizobacteria With Plant Growth-Promoting Traits

Based on previous evidence of fertilization altering the structure of wheat rhizosphere bacterial communities (Kavamura et al., 2018), we hypothesized that addition of fertilizer would reduce, specifically, the presence of putative PGPR.

**FIGURE 4:**
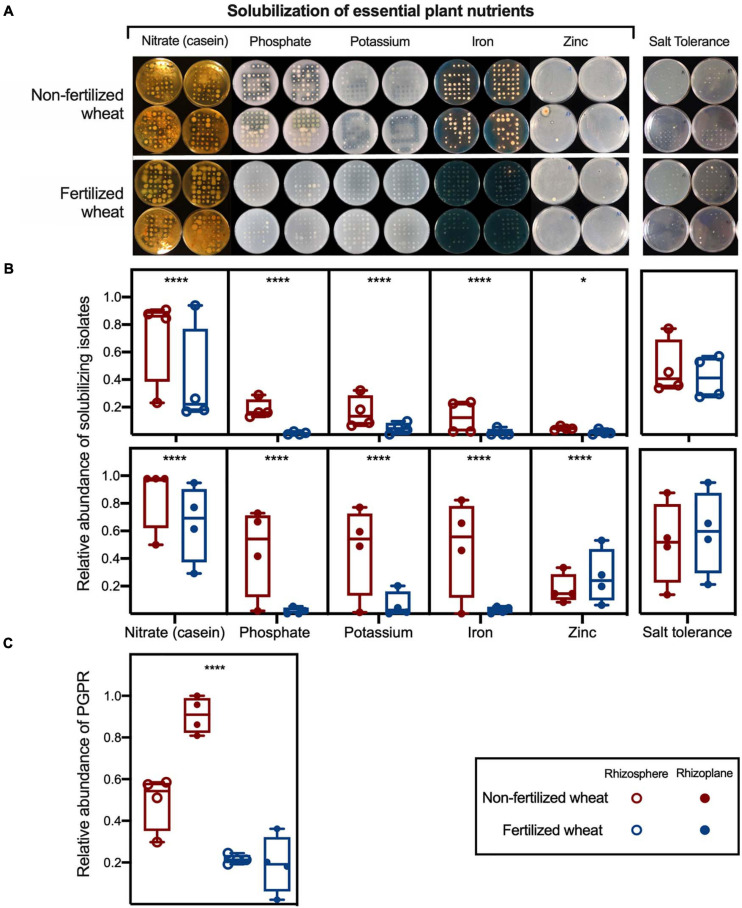
Relative abundance of culturable rhizobacteria with plant growth-promoting traits isolated from wheat grown in soils with and without fertilizer. **(A)** Nitrate-, phosphate-, potassium-, iron-, and zinc-solubilization as determined by casein, PVK, AVK, CAS, and zinc carbonate agar, respectively. Casein, phosphate, potassium, and zinc solubilization are indicated by clear halos surrounding the colony whereas orange halos indicate iron solubilization. Salt tolerance as determined by 10TSA supplemented with 5% NaCl. Box plots show relative abundance of **(B)** solubilizing isolates and salt tolerant isolates and **(C)** relative abundance of PGPR isolates, as determined by functional bioassays; with median (line) and hinges at first and third quartiles (25th and 75th percentiles). Each point represents an isolate library (*n* = 94) derived from rhizosphere (open circles) and rhizoplane (closed circles) samples. Single colonies were isolated from different non-selective agar and 10TSA respectively and inoculated in TSB (1/10th concentration) in a 96-well plate; sterile TSB (1/10th concentration) in 2× wells were left uninoculated to act as a negative control. Significant differences as determined by Chi-squared tests are shown by “*” and “****” for *p* < 0.05 and *p* < 0.0001, respectively.

In general, rhizobacteria that tested positive for solubilization of plant macronutrients: organic N (casein), inorganic phosphate [Ca_3_(PO_4_)_2__]_ and potassium (potash feldspar), and plant micronutrients: iron (FeCl_3_•6H_2_O) and zinc (ZnO), had a statistically higher relative abundance in isolate libraries cultured from non-fertilized wheat samples compared to fertilized wheat samples (Figures 4A,B; Supplementary Table 9). However, there was no significant difference in the relative abundance of salt-tolerant rhizobacteria in non-fertilized wheat samples compared to fertilized wheat samples (Figure 4B; Supplementary Table 9). Relative abundance of nutrient solubilizing isolates were greater in rhizosphere (49.2 ± 13%; two-tailed Chi-square test: χ^2^ = 116, *p* < 0.0001, d.f. = 1) and rhizoplane (90.7 ± 9%; χ^2^ = 389, *p* < 0.0001, d.f. = 1) samples from non-fertilized wheat compared to fertilized wheat (21.5 ± 2 and 19.1 ± 14% from rhizosphere and rhizoplane, respectively) (Figure 4C).

**FIGURE 5:**
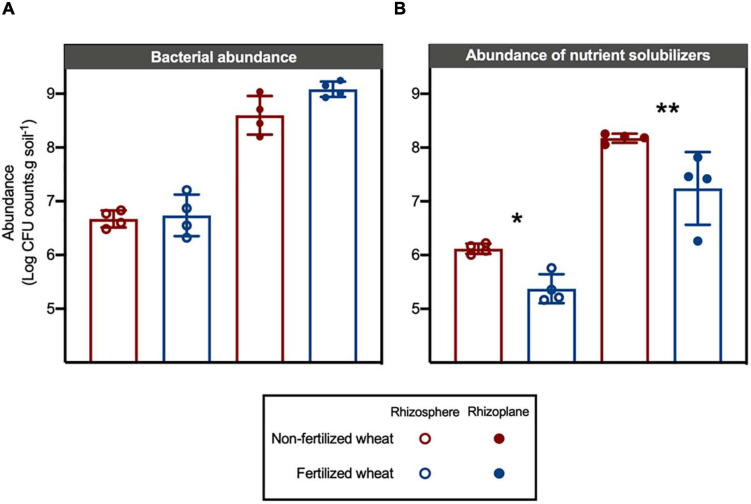
Effect of chemical fertilizer (NPK) on absolute abundance of culturable bacteria isolated from the rhizosphere and rhizoplane of wheat grown in low nutrient soil. **(A)** Absolute abundance (log CFU counts.g soil^–1^; CFU is colony-forming units) of culturable rhizobacteria isolated from soil samples on 10TSA. **(B)** Absolute abundance (log CFU counts.g soil^–1^) of nutrient solubilizing rhizobacteria as determined by functional bioassays. Significant differences as determined by *post hoc* multiple comparison tests with Šidák correction are shown by “*” and “**” for *p* < 0.05 and *p* < 0.01, respectively.

Absolute abundance of bacteria isolated from the rhizoplane (mean log CFU counts.g soil^–1^: 8.84 ± 0.34) was higher compared to the rhizosphere of wheat (mean log CFU counts.g soil^–1^: 6.70 ± 0.05) (two-way ANOVA: *F* = 18.3, d.f. = 1, *p* < 0.0001) whereas fertilizer addition had no significant effect on rhizobacterial abundance (*p* > 0.05) (Figure 5A). In comparison, absolute abundance of nutrient-solubilizing bacteria isolated from rhizocompartments in wheat were statistically higher from rhizoplane samples (*F* = 113, d.f. = 1, *p* < 0.0001) and were also statistically higher in non-fertilized compared to fertilized wheat samples (*F* = 20.7, d.f. = 1, *p* = 0.0007) (Figure 5B). *Post hoc* multiple comparison tests with Šidák correction showed differences in means in rhizosphere (log CFU counts.g soil^–1^: 8.18 ± 0.08 and 7.24 ± 0.67 in non-fertilized and fertilized wheat, respectively) (*p* = 0.0289) and rhizoplane (mean log CFU counts.g soil^–1^: 6.12 ± 0.09 and 5.37 ± 0.27 in non-fertilized and fertilized wheat, respectively) (*p* = 0.0075) samples. However, there were no statistical differences between individual assays for absolute abundance of isolates from non-fertilized vs. fertilized wheat (Supplementary Figure 6).

### Identification of Culturable Isolates Within Culture-Independent and -Dependent Amplicon Datasets

**FIGURE 6:**
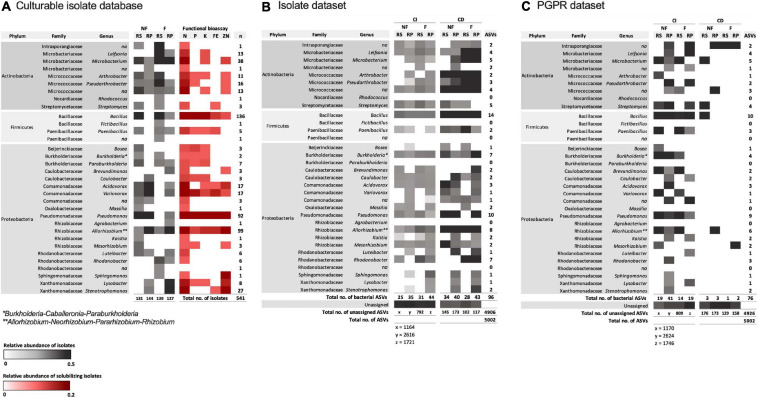
Heatmaps representing abundance and associated functional ability of bacterial isolates from rhizosphere (RS) and rhizoplane (RP) samples from non-fertilized (NF) and fertilized (F) wheat. **(A)** The full region of the 16S rRNA gene was amplified and sequenced for each rhizobacterial isolate. Identifications are based on the SILVA database for prokaryotes. Gray indicates relative abundance of genera in each sample (number of isolates per sample shown below columns). Red indicates relative abundance of positively solubilizing isolates from total number of isolates sequenced (541). “*n*” shows number of genera identified from total number of isolates sequenced. **(B)** All culturable rhizobacterial isolate sequences and **(C)** plant growth-promoting rhizobacteria (PGPR) isolate sequences were used to create a database with which the culture-independent (CI) and culture-dependent (CD) amplicon datasets were searched against using blast at 100% sequence identity. Burkholderia* stands for Burkholderia-Caballeronia-Paraburkholderia; Allorhizobium** stands for Allorhizobium-Neorhizobium-Pararhizobium-Rhizobium.

An isolate database was curated from 541 isolates (275 from non-fertilized wheat; 266 from fertilized wheat) which consisted of a total of 27 genera (Figure 6; Supplementary Data 1). Rhizobacterial isolates that displayed growth-promoting abilities were identified as being mostly *Bacillus* species (48%) in the rhizosphere and *Pseudomonas* (50%) in the rhizoplane of non-fertilized wheat (Figure 6A). For fertilized wheat plants the majority of isolates were also identified as *Bacillus* (52%) in the rhizosphere and *Allorhizobium-Neorhizobium-Pararhizobium-Rhizobium* (48%) in the rhizoplane (Figure 6A).

In total, 96 (1.92%) isolate bacterial ASVs were also identified as being present in the total community amplicon 16S rRNA gene dataset from this work. The remaining 4,906 ASVs could not be assigned to any culturable isolates from this work; 88 (1.84%) bacterial ASVs were identified in the CI amplicon dataset and 58 (13.6%) bacterial ASVs were identified in the CD amplicon dataset (Figure 6B). Comparatively, at 99% sequence identity, 246 (4.5%) isolate ASVs were classified as bacteria from 5,456 ASVs. However, to increase accuracy of isolate identification, one hundred percent sequence identity was used. Overall, 23 genera were identified in the CI and CD amplicon datasets, when searched against the isolate database; no ASVs were classified as *Rhodococcus, Fictibacillus, Paraburkholderia*, and *Agrobacterium*.

**FIGURE 7:**
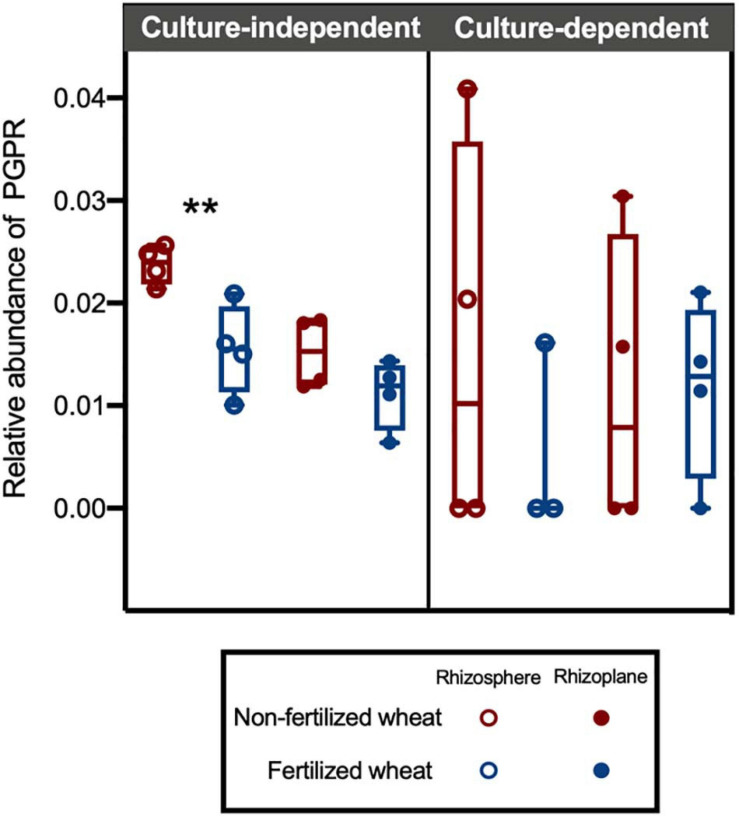
Box plots showing relative abundance of ASVs identified as putative plant growth-promoting rhizobacteria (PGPR) in culture-independent and -dependent isolate datasets. A PGPR database was created from 16S rRNA gene sequences from culturable bacterial isolates with acquisitional traits for key plant nutrients. Each point represents the proportion of ASVs that were identified as Bacteria in CI and CD datasets, with median (line) and hinges at first and third quartiles (25th and 75th percentiles). Significant differences as determined by *post hoc* multiple comparison tests with Šidák correction are shown by “**” for *p* < 0.01.

The PGPR isolate database identified 76 ASVs (1.52%) as PGPR within the CI and CD communities (Figure 6C). Overall, relative abundance of PGPR isolates were relatively low (<5%) however, there was a statistically higher mean abundance of PGPR in non-fertilized CI samples (0.019 ± 0.006) compared to fertilized CI samples (0.012 ± 0.002) (two-way ANOVA: *F* = 22.7, d.f. = 1, *p* = 0.0005) (Figure 7). Compartment also contributed to difference in means and was statistically higher in the rhizosphere (0.020 ± 0.006) compared to the rhizoplane (0.013 ± 0.003) (two-way ANOVA: *F* = 14.7, d.f. = 1, *p* = 0.0024). There was no significant interaction with fertilization regime. *Post hoc* multiple comparison tests with Šidák correction showed differences in means in rhizosphere (0.024 ± 0.002 and 0.016 ± 0.004 in non-fertilized and fertilized wheat, respectively) (*p* = 0.0009) but not in rhizoplane (0.015 ± 0.003 and 0.011 ± 0.003 in non-fertilized and fertilized wheat, respectively) (*p* > 0.05) samples.

### Major ASVs Within Wheat Rhizobacterial Communities

**FIGURE 8:**
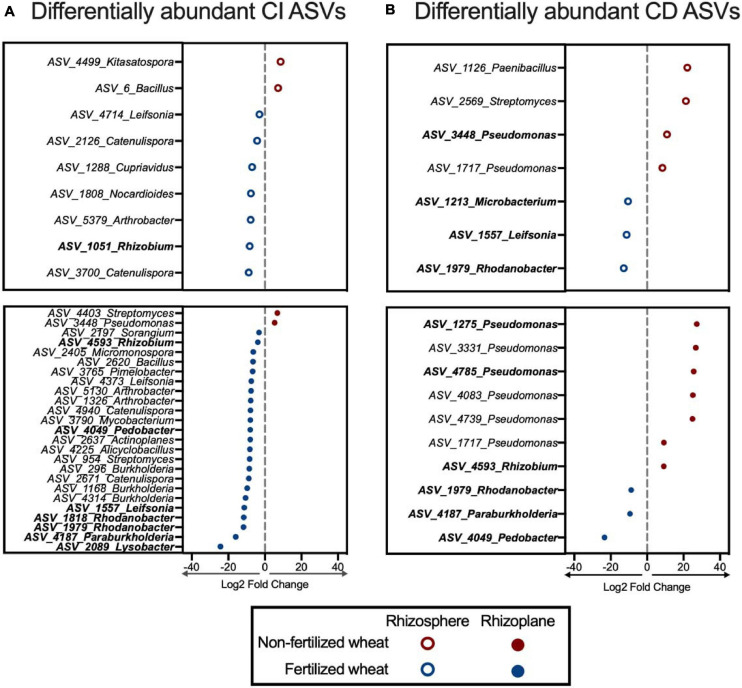
ASVs that significantly differ in abundance in non-fertilized wheat vs. fertilized wheat. Differentially abundant ASVs detected by DESeq2 at a significance level of *p*_–__*adjusted*_ < 0.05 which were found to be enriched in **(A)** culture-independent (CI) and **(B)** culture-dependent (CD) rhizosphere (open circles) and rhizoplane (closed circles) communities in non-fertilized and fertilized wheat. ASVs depicted in bold were also detected in the isolate dataset. Only ASVs classified to genus level are pictured; for full list see Supplementary Material.

Differential abundance analysis identified ASVs that were highly associated with non-fertilized wheat compared to fertilized wheat in rhizosphere and the rhizoplane samples. Overall: 54 ASVs (3.5% of all ASVs identified in CI rhizosphere samples); 115 ASVs (3.5% of all ASVs identified in CI rhizoplane samples); 17 ASVs (6.4% of all ASVs identified in CD rhizosphere samples); and 16 ASVs (6.0% of all ASVs identified in CD rhizoplane samples) were differentially abundant between non-fertilized and fertilized samples (Supplementary Tables 7, 8). Six genera (15 ASVs) were enriched in non-fertilized samples (*p* < 0.01) and they included members of the Proteobacteria (46.97%), Firmicutes (9.09%), and Actinobacteria (7.58%) (Figure 8). Conversely, 19 genera (36 ASVs) were enriched in fertilized samples and they included members of the Proteobacteria (49.26%), Actinobacteria (31.62%), Bacteroidetes (10.29%), and Firmicutes (4.41%) (Figure 8). In particular nine ASVs identified as *Pseudomonas* were enriched in non-fertilized samples compared to fertilized samples where four ASVs identified as *Catenulispora, Leifsonia*, and *Rhodanobacter* were enriched (Figure 8).

Key ASVs that were differentially abundant in both the amplicon dataset and isolate dataset were identified (*p* < 0.01). They included: *ASV_3448_Pseudomonas*, *ASV_1275_Pseudomonas*, and *ASV_4785_Pseudomonas* in non-fertilized wheat samples and *ASV_1051_Rhizobium*, *ASV_4593_Rhizobium*, *ASV_4049_Pedobacter*, *ASV_1557_ Leifsonia*, *ASV_1818_Rhodanobacter*, *ASV_1979_Rhoda nobacter*, *ASV_4187_Paraburkholderia*, and *ASV_2089_ Lysobacter* in fertilized wheat samples (Figure 8). Four ASVs (ASV_4593, ASV_4049 and ASV_1979, ASV_4187) were differentially more abundant in fertilized wheat samples in CI and CD amplicon datasets and CI and CD isolate datasets.

## Discussion

We hypothesized that the addition of chemical fertilizer would reduce putative PGPR populations in wheat. We found that the abundance of rhizobacteria with acquisitional traits for key plant nutrients (endogenous nitrogen, phosphate, potassium, iron, and zinc mobilization) were significantly reduced in wheat grown in soils treated with NPK fertilizer.

We combined both CI and -dependent methods to study the impact of fertilizer on microbiome community composition and diversity. Our CI results confirmed previous studies showing that fertilizer alters community structure and reduces bacterial alpha diversity in the root environment (Jorquera et al., 2014; Zhu et al., 2016; Cui et al., 2018; Kavamura et al., 2018; Lian et al., 2018; Chen et al., 2019; Pagé et al., 2019; Liang et al., 2020). Our CD results support this as beta diversity was influenced and alpha diversity reduced by fertilizer. We decided to analyze CI rhizosphere and rhizoplane datasets together despite their having been sequenced using different approaches because, whilst inter-sequencing center variation can be significant (Schloss et al., 2011), treatment effect consistently outweighs run variation (Wen et al., 2017). Thus, we acknowledge that compartment effect (in CI samples) could be partially due to bias in sequencing run; however, it is unlikely only due to this given the high percentage variability in beta-diversity, and previous evidence that different soil compartments have distinct microbial compositions (van der Heijden and Schlaeppi, 2015). Additionally, our main aim was to study the effect fertilizer had on rhizobacterial diversity which is distinct in both compartments. Finally, we have previously found that fertilizer application reduces microbial species richness in the rhizosphere (Kavamura et al., 2018) and when re-examining the dataset from this study there is also a reduction in species richness in the bulk soil of plots receiving high levels of inorganic N fertilizer, but this effect is exacerbated in the rhizosphere. Therefore, we conclude that despite fertilizer also influencing bacterial community structure in bulk soil (Chen et al., 2016; Ding et al., 2016; Francioli et al., 2016; Soman et al., 2017; Kumar et al., 2018; Dai et al., 2020) it also has a profound effect on the rhizo-microbiome.

We tested how much the use of multiple agar types increased the percentage of total community DNA retrieved when compared to commonly used 10TSA only. The agar types were selected from Bai et al. (2015) which characterized extensive culture collections isolated from *Arabidopsis* leaf and root microbiomes and demonstrated that the majority of leaf- and root-dwelling microbes were amenable to culture. Whilst Bai et al. (2015) used colony picking, as well as limiting dilution and cell sorting to purify bacterial isolates we were more interested in mixed culture taxonomy that resulted from the use of different isolation media, similar to the method used in Kavamura et al. (2019). In fact, the use of seven additional agar types increased the percentage of ASVs retrieved twofold compared to 10TSA only which, considering 10TSA retrieved half the ASVs from all agar types combined, supports its representability as an isolation medium. We reported a higher percentage of culturable ASVs (4.2%) retrieved in total community DNA, in contrast to Stefani et al. (2015) and Kavamura et al. (2019) who retrieved 2.4 and 2.2%, respectively, using similar isolation methods. Both studies analyzed 16S rRNA gene datasets by clustering sequences into operational taxonomic units; the increased percentage retrievability which we report could be due to improved taxonomic resolution from ASV analysis (Fierer et al., 2017). Additionally, we found that agar type had little effect on the diversity and composition of rhizobacterial isolates cultured from soil. We conclude that this is likely due to the agar types used being less selective, not genera-specific and similar in composition which would explain the lack of diversity yielded. It would be interesting in future work to test microbial composition isolated from agar with similar conditions as found in soil, such as soil extract, root exudate, and plant extract media.

Putative PGPR were characterized using a variety of functional bioassays to test a representative population of the culturable rhizo-microbiome. We found a clear difference in relative abundance of nutrient-solubilizing bacteria isolated from rhizosphere and rhizoplane samples between fertilized and non-fertilized wheat. There was a marked reduction in both relative and absolute abundance of nutrient solubilizing bacteria in fertilized samples. We hypothesized that this would be the case since fertilized plants and microorganisms can utilize easily available NPK source and do not need to solubilize NPK. Indeed, key enzymes involved in microbial solubilization of P, alkaline phosphatases, were shown to be strongly decreased by P fertilization in the rhizosphere (Spohn and Kuzyakov, 2013; Widdig et al., 2019) which suggests that fertilizer reduces hydrolytic enzyme-producing microorganisms or enzyme production in organisms capable of such function. The role of fertilization on soil enzymatic activities has been investigated in detail over the last 40 years (Bautista-Cruz and Ortiz Hernandez, 2015) where increasing evidence suggests that chemical fertilization can inhibit or slow down synthesis of hydrolytic enzymes. However, linking enzyme production to individual microbiome members or groups is particularly challenging (Sergaki et al., 2018). Our study suggests that genera such as *Bacillus, Pseudomonas, Rhizobium*, and *Streptomyces* are producers of hydrolytic enzymes in soil. Of course, this study does not exclusively show that the culturable rhizobacteria with plant growth-promoting traits contributed to plant growth, and it is possible that the bacteria were releasing nutrients for their own consumption. However, spatial differentiation of microbes in the rhizosphere has been demonstrated to reduce plant-microbe competition as much as possible (Marschner et al., 2011). Additionally, taxonomically and functionally similar bacteria to those we isolated in this study have been shown to increase plant growth when inoculated in soils (Assainar et al., 2018; Masters-Clark et al., 2020; Wang et al., 2020; Wilkinson, 2020).

We hypothesized that there is a host selection process for nutrient-solubilizing bacteria, driven by poor nutrient availability conditions and that fertilizer reduces this selection as wheat can utilize the readily available nutrients in soil and no longer need to interact with beneficial bacteria. Our results show a higher abundance of nutrient-solubilizing rhizobacteria in the rhizoplane, a compartment more intimately associated with the plant host than the rhizosphere, which could suggest a plant mediated selection process. Alternatively, the competition for nutrients might be heightened in the rhizoplane as compared to the rhizosphere, and nutrient-solubilizing organisms could be under a stronger selection when nutrients are depleted. We also found bacteria with known plant growth-promoting properties such as *Paenibacillus, Streptomyces*, and *Pseudomonas* to be more abundant in root-associated soil from non-fertilized wheat (Vejan et al., 2016; Liu et al., 2019). However, it is unclear why fertilizer addition would inhibit root colonization by these bacteria. It is possible that rhizobacteria are less able to metabolize primary nutrients in the form presented in agricultural fertilizers than other members of the soil microbiome. If this is the case, it would follow that they are also less competitive in this environment and this would be reflected in their lower abundance. *Pseudomonas*, for example, colonize plants through chemotaxis into the rhizosphere along a gradient of root exudates, followed by surface association and migration on the rhizoplane to ultimately form a bacterial biofilm, which would explain their increased abundance in the rhizoplane. In addition, many *Pseudomonas* spp. produce enzymes and other signaling molecules such as lipopolysaccharides to successfully colonize the plant rhizosphere and manipulate plants by activating symbiotic pathways (Oldroyd, 2013). It has been shown that nutrient starvation triggers plants to activate symbiotic pathways with bacteria (Zipfel and Oldroyd, 2017), so addition of fertilizer could mean plants no longer need to activate such pathways and thus no longer interact with beneficial bacteria, such as *Pseudomonas*. Chen et al., 2019 showed that the order Pseudomonadales in rhizosphere samples responded negatively to organic acid released by wheat in response to N fertilization (Chen et al., 2019) which correlate with our results that show a reduced abundance of *Pseudomonas* in soil samples from fertilized wheat and suggest that organic acid release by wheat as a response to fertilizer addition indirectly inhibits selection and colonization of *Pseudomonas* of the root system. The higher abundance of pseudomonads was more apparent in culturable rhizobacterial communities. Other non-culturable species may play just as important a role but are not as amenable to study.

Reducing environmental and financial costs of conventional agriculture requires novel management and breeding strategies to shift current high input to more sustainable biological methods. Soil microbiome manipulation promises to contribute to more environmentally benign agriculture by promoting plant growth and suppressing pathogenic microorganisms (Mauchline and Malone, 2017) and show promise in reducing chemical fertilizer application without influencing crop growth (Assainar et al., 2018; Wang et al., 2020). Our work provides a binary method to determine abundance of bacteria with beneficial traits in soil samples. Furthermore, we endeavored to link individual isolates cultured from soil samples to total soil community DNA. Culturable bacteria with plant growth-promoting traits are invaluable to the commercialization of biostimulants for sustainable agriculture (Backer et al., 2018). Therefore, establishing their abundance within total soil DNA will be useful to determine the overall impact that bioinoculants have on the microbiome as well as to determine optimal inoculation dose. However, first we must understand plant host evolutionary and domestication history to identify plant traits linked to microbial recruitment by wild relatives (Perez-Jaramillo et al., 2016). Significant knowledge gaps limit our understanding of the soil microbiome. The field needs to move beyond simple descriptions of community diversity to identify patterns in this complexity and recognize when that complexity is important (Lemanceau et al., 2017). This will enable us to make soil microbiome research of practical utility to human undertakings, toward a cooperative relationship with terrestrial ecosystems instead of our current one-sided and often self-destructive relationship.

This study contributes to our understanding of the impact of fertilizer on wheat rhizobacteria and supports previous studies showing the deleterious effect of chemical fertilizer on plant rhizobacteria, particularly through highlighting the greater abundance of putative PGPR in unfertilized plants. It is assumed that wild relatives have co-evolved with the microbial community of native soils, selecting microbes beneficial to growth and health. Here, we show the probability that wheat plants can select growth-promoting bacteria to their roots to establish mutually beneficial associations and that chemical fertilizer reduces this selection. We propose a CD method to characterize the functional ability of the microbiome, and link this to CI total soil DNA. We hope that this work contributes to the shift from simple microbiome studies on taxonomic characterization to conceptual framework analysis identifying and explaining patterns in the soil microbiome in agriculture and natural systems (Harris, 2009).

## Data Availability Statement

The raw sequences analyzed for this study can be found in the NCBI Sequence Read Archive (SRA), accession PRJNA625513 and in NCBI Genbank, accessions MT354024 – MT354564.

## Author Contributions

TR, TM, IC, and VK designed the experiments. TR, VK, and MA performed the experiments and collected the data. TR analyzed the data with input from AT-B, VK, and MA for bioinformatic analyses. TR wrote the manuscript. All co-authors edited and commented on the manuscript.

## Conflict of Interest

The authors declare that the research was conducted in the absence of any commercial or financial relationships that could be construed as a potential conflict of interest.

## Footnotes

1 http://www.r-project.org

2 www.arb-silva.de/act
