# Supplementary material for: Inorganic Chemical Fertilizer Application to Wheat Reduces the Abundance of Putative Plant Growth-Promoting Rhizobacteria
Source: Front Microbiol. 2021 Mar 11;12:642587. doi: 10.3389/fmicb.2021.642587 (PMC7991844; doi:10.3389/fmicb.2021.642587)
Supplement: Supplementary file 1 [file Data_Sheet_1.zip › Supplementary_material/Supplementary_material.pdf]

Supplementary material

Inorganic Chemical Fertilizer Application to Wheat Reduces the Abundance of Putative Plant Growth-Promoting Rhizobacteria

Tessa E. Reid[1 2], Vanessa N. Kavamura[1], Maïder Abadie[1], Adriana Torres-Ballesteros[1], Mark Pawlett[2], Ian M. Clark[1], Jim Harris[2] and Tim H. Mauchline[1*]

[1]Sustainable Agriculture Sciences, Rothamsted Research, West Common, Harpenden, AL5 2JQ, UK

[2]Cranfield Soil and Agrifood Institute, Cranfield University, College Road, Cranfield, Bedford, MK43 0AL, UK

***Correspondence:**
Tim H. Mauchline
tim.mauchline@rothamsted.ac.uk

SUPPLEMENTARY METHODS

Amplicon library preparation and sequencing

All PCR reactions were conducted with the KAPA HiFi HotStart ReadyMix PCR Kit (Kapa, Wilmington, MA, USA) (0.5 µM each primer, 3 µl template DNA (150 ng), 30 µl reaction volume, 96-well skirted PCR plates (StarLab)). PCR amplifications were performed with initial denaturation at 95°C (3 min) followed by 30 cycles at 95 °C (20 s), 55 °C (15 s), and 72 °C (20 s), and a final elongation step at 72 °C (1 min). Amplification reactions were verified by agarose gel electrophoresis and purified using a magnetic bead capture kit (Ampure; Agencourt). The resulting indexed amplicons were quantified on a Qubit Fluorimeter using the Qubit dsDNA High Sensitivity assay (Thermo Scientific) and normalized to equimolar concentration prior to being pooled. The final pool was quantified, and quality assessed using SYBR green quantitative PCR (qPCR) assay with primers specific to the Illumina adapters (Kappa) and diluted to 12 pM. The library was spiked with PhiX control V3 library (5% v, v^{-1}, 12.5 pM) and sequenced on the Illumina MiSeq platform using the Illumina MiSeq v3 kit (Illumina Inc.), to obtain 2 x 300 bp paired-end sequences.

Colony PCR

Bacterial isolates were spot inoculated on 10TSA from frozen glycerol stocks, using a 48-prong manifold, and grown at 25 °C for 2 days. A sterile toothpick was used to inoculate a single isolate in 40 µl lysis buffer (Tris-EDTA, 0.1% Triton) in a skirted 96-well PCR plate (StarLab, Milton Keynes, UK). Bacterial suspensions were vigorously vortexed, then boiled at 100 °C in a PCR Thermal Cycler (MJ Research PTC-200) for 2 min before being frozen at -80 °C for 30 min. An aliquot of the resulting solution was used for colony PCR. All PCR reactions were conducted with the DreamTaq Green PCR Master Mix (2x) (Thermo Scientific), 1 µM of each primer, and 2.5 µl bacterial lysis solution in a 25 µl reaction volume in 96-well non-skirted PCR plates (StarLab). PCR amplifications were performed with an initial denaturation at 95°C (5 min) followed by 35 cycles at 95 °C (30 s), 60 °C (30 s), and 72 °C (1 min), and a final elongation step at 72 °C (5 min). Amplification reactions were verified by agarose gel electrophoresis and sent to Eurofins Genomics Germany for purification and Sanger sequencing.

SUPPLEMENTARY FIGURES

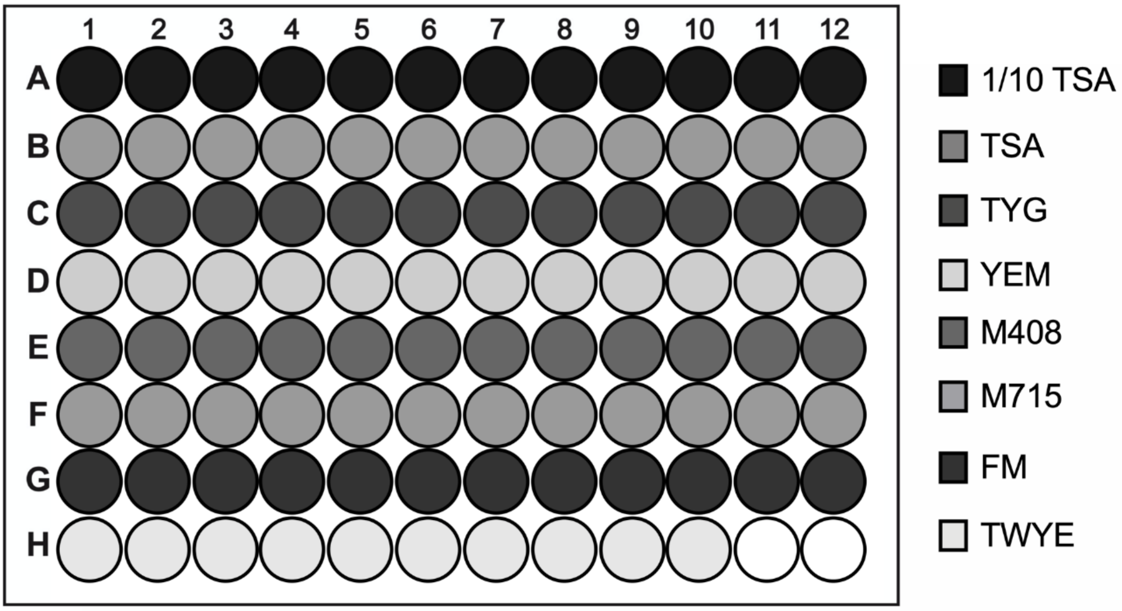

Supplementary Figure S1. Schematic representation of 96-well plate design for isolate library. Sterile tryptone soya broth (TSB; at 1/10th conc.; 500 µl) was used as a negative control (Wells H11 and H12).

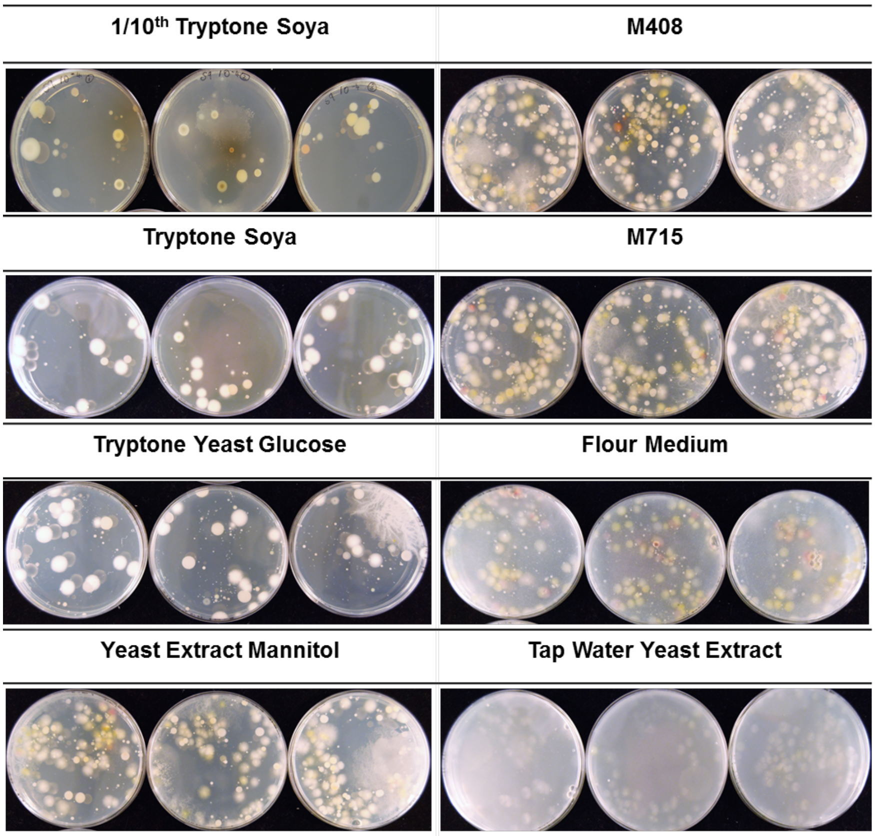

Supplementary Figure S2. Rhizobacterial isolates cultured on agar for taxonomic analysis. Samples plated on 1/10th tryptone soya, tryptone soya, tryptone yeast glucose, yeast extract mannitol, m408 and m715 were at 10^{-3} dilution; samples plated on flour medium and tap water yeast extract were at 10^{-2} dilution.

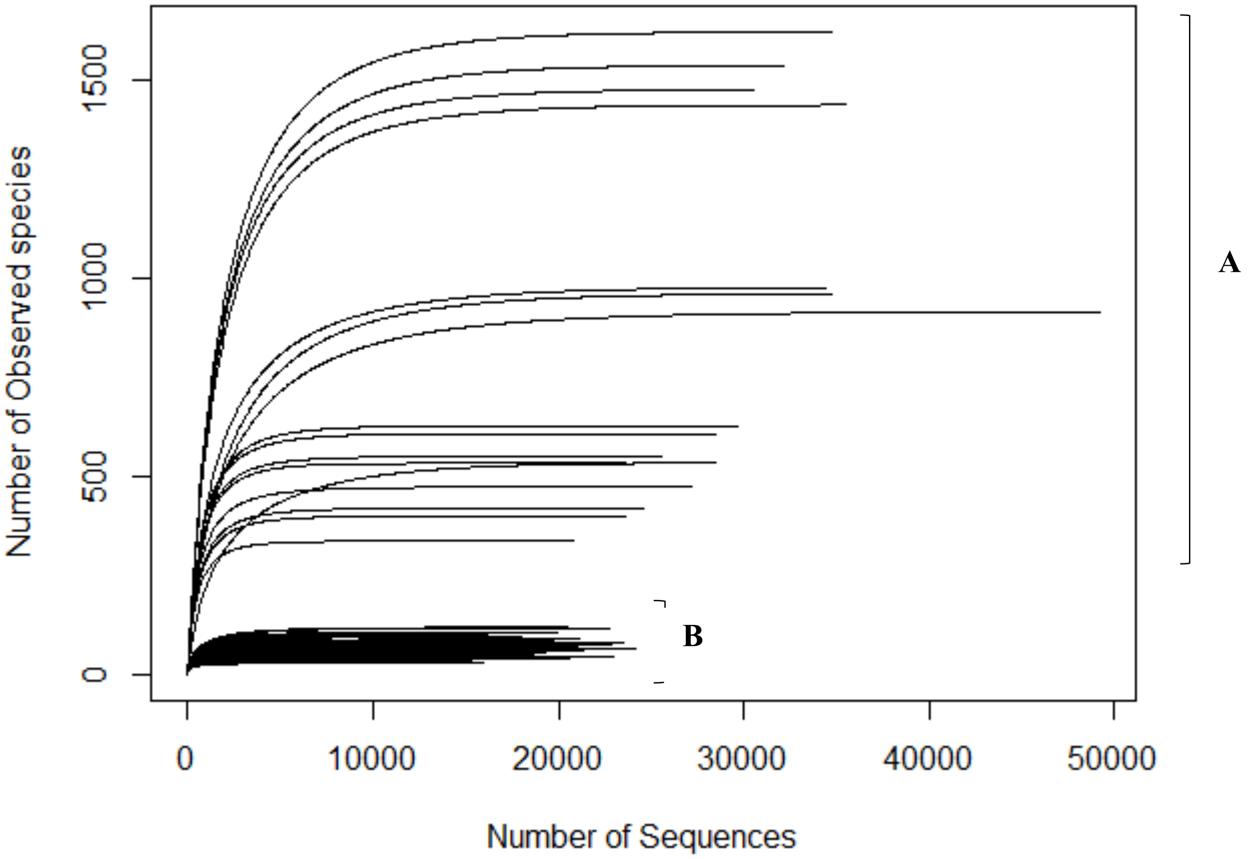

Supplementary Figure S3. Rarefaction curve analyses of bacterial species richness as a function of sequencing depth. Culture-independent sequences (A) and culture-dependent sequences (B).

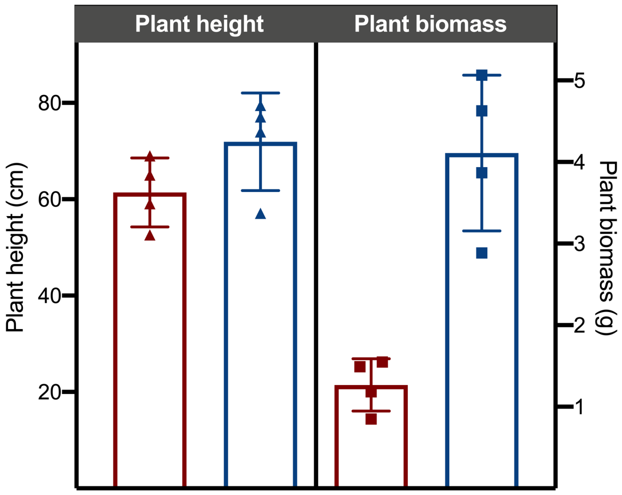

Supplementary Figure S4. Plant height and dry biomass of wheat grown in soils with and without fertilizer addition

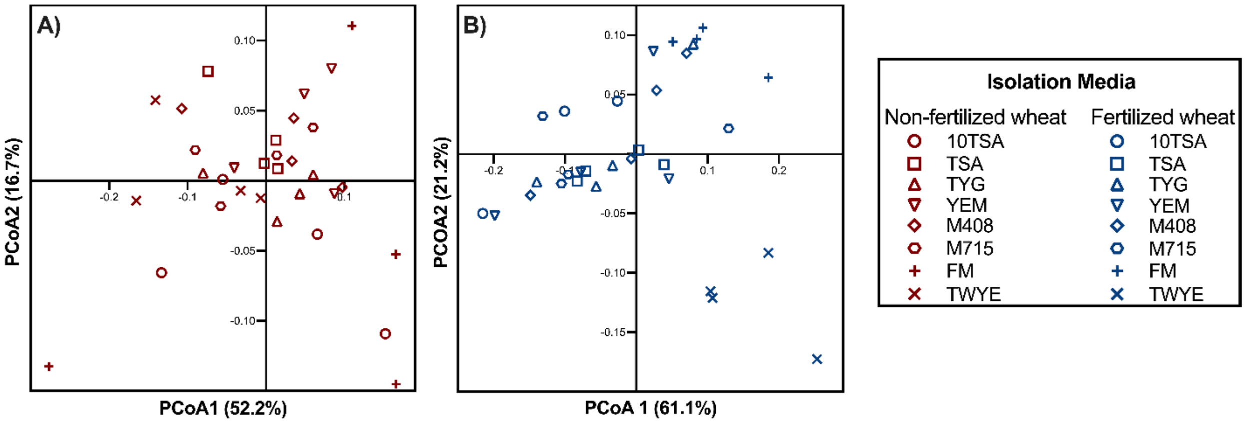

Supplementary Figure S5. PCoA plots of rhizosphere bacteria cultured on a variety of agar types based on weighted UniFrac distances at ASV level, from (A) non-fertilized wheat and (B) fertilized wheat.

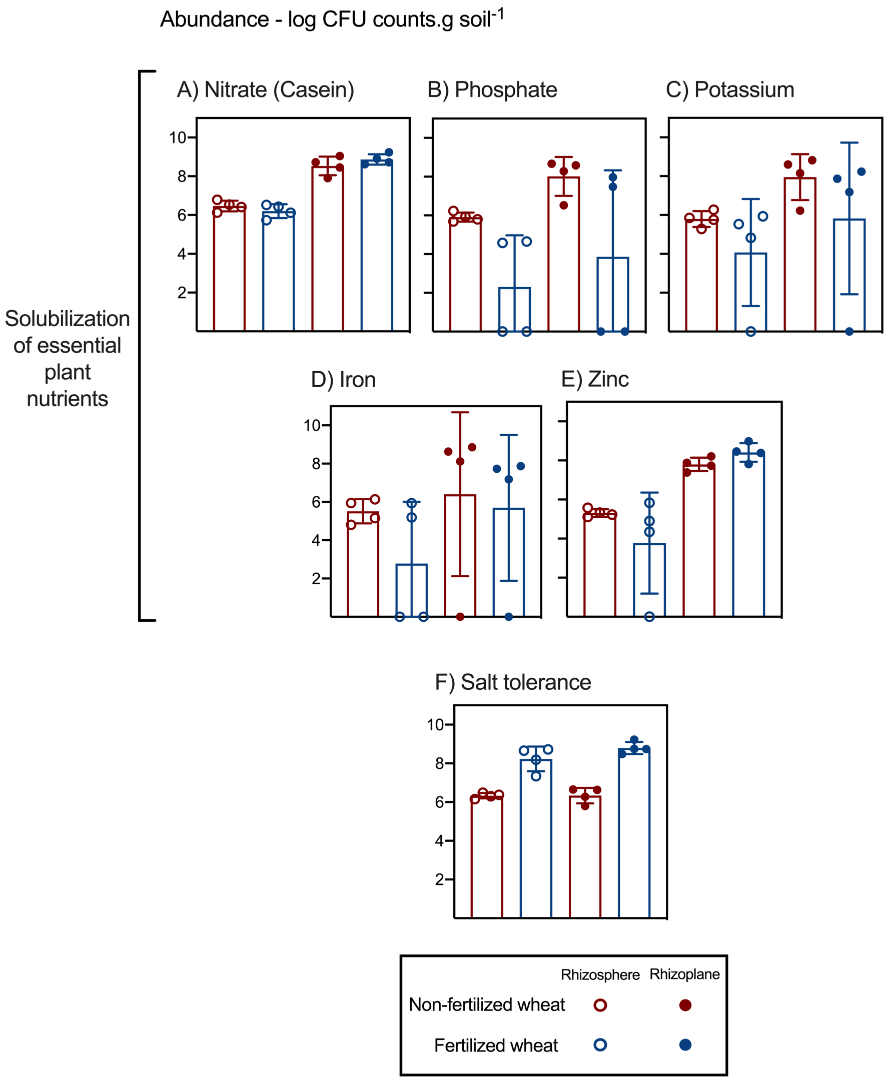

Supplementary Figure S6. Absolute abundance of culturable rhizobacteria with plant growth-promoting traits isolated from wheat grown in soils with and without fertilizer.

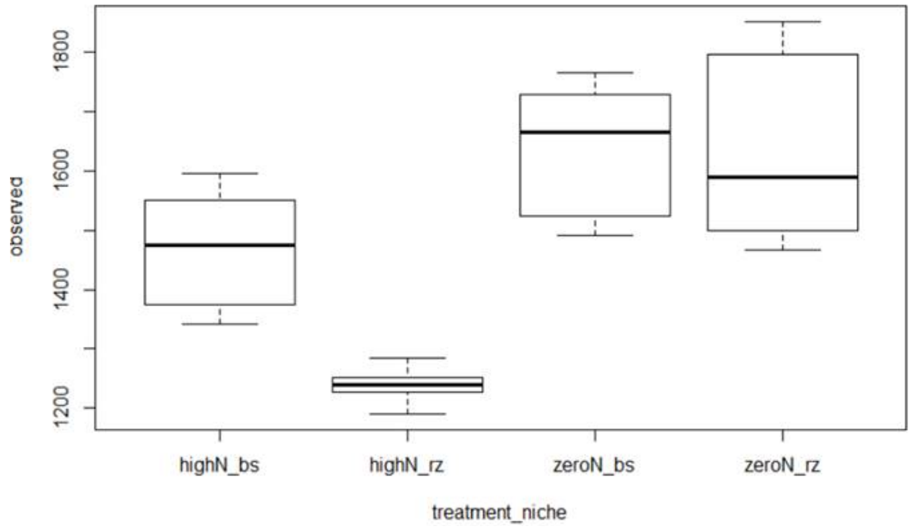

Supplementary Figure S7. Observed operational taxonomic units (OTUs) within bacterial 16S V4 sequences from bulk soil (bs) and wheat rhizosphere (rz) samples with (highN) and without fertilizer (zeroN) addition. Reanalyzed data from Kavamura et al., 2018 (1).

SUPPLEMENTARY TABLES

Supplementary Table S1. Composition of bacterial growth agar used for isolation approaches

Compound	Tryptone Soy Broth (TSA)	Tryptone Yeast Extract Glucose (TYG)	Yeast Extract Mannitol (YEM)	M408	M715	Flour Medium (FM)	Tap Water Yeast Extract (TWYE)
	gram per litre (g/l) of MilliQ water						gram per litre (g l/l) of tap water
Casein (pancreatic digest)	17.0	x	x	x	x	x	x
Casein (enzymatic hydrolysate)	x	5.0	x	x	x	x	x
Soya peptone (papaic digest)	3.0	x	x	x	x	x	x
NaCl	5.0	x	x	x	x	x	x
K_2HPO_4	2.5	x	x	x	x	x	0.5
Dextrose	2.5	x	x	x	x	x	x
D-glucose	x	1.0	x	x	x	x	x
Yeast extract	x	3.0	0.5	1.0	1.0	x	0.3
Mannitol	x	x	5.0	10.0	10.0	x	x
K_2HPO_4	x	x	0.5	0.5	0.5	x	x
$MgSO_4 \cdot 7H_2O$	x	x	0.2	0.2	0.2	x	x
NaCl	x	x	0.1	0.1	0.1	x	x
$CaCO_3$	x	x	x	x	1.0	0.4	x
Plain flour	x	x	x	x	x	4.0	x
Agar	20	15	20	20	20	16	18
pH	7.0	7.0	7.0	7.0	7.0	7.0	7.0

Supplementary Table S2. Outline of technical replicates plated per soil sample on agar

Selective Agar	Dilution x10-2	Dilution x10-3	Dilution x10-4
10TSA	NA	3x	3x
TSA	NA	6x	NA
TYG	NA	6x	NA
YEM	3x	3x	NA
M408	3x	3x	NA
M715	3x	3x	NA
FM	6x	NA	NA
TWYE	6x	NA	NA

Supplementary Table S3. PERMANOVA of culture-independent rhizobacterial beta-diversity as represented by weighted UniFrac metric. This is a two-way PERMANOVA analysis with 999 permutations.

Sources of Variation	Degrees of Freedom	Sum of Squares	F statistic	R2	Pr(>F)	Statistical Significance
Rhizocompartment	1	0.0155	16.066	0.3597	0.001	***
Fertilizer	1	0.01233	13	0.2861	0.001	***
Fertilizer: Rhizocompartment	1	0.00369	3.825	0.0856	0.014	*
Residuals	12	0.01158				

Significance codes: 0.0001'***' 0.001 '**' 0.01 '*' 0.05 '.' 0.1 ' ' 1

Supplementary Table S4. PERMANOVA of culture-dependent rhizobacterial beta-diversity as represented by weighted UniFrac metric. This is a two-way PERMANOVA analysis with 999 permutations.

Sources of Variation	Degrees of Freedom	Sum of Squares	F statistic	R2	Pr(>F)	Statistical Significance
Rhizocompartment	1	0.3188	6.223	0.2948	0.002	**
Fertilizer	1	0.1562	3.0479	0.1444	0.035	*
Fertilizer: Rhizocompartment	1	0.04282	0.8358	0.0396	0.474	
Residuals	11	0.5636				

Significance codes: 0.0001 '***' 0.001 '**' 0.01 '*' 0.05 '.' 0.1 ' ' 1

Supplementary Table S5. ANOVA of culture-independent rhizobacterial alpha diversity as represented by the Shannon diversity index. This is a two-way type III ANOVA.

Sources of Variation	Degrees of Freedom	Sum of Squares	F statistic	Pr(>F)	Statistical Significance
Fertilizer	1	5.074	14.62	0.0024	**
Fertilizer: Rhizocompartment	1	1.566	4,512	0.05512	.
Rhizocompartment	1	0.312	0.899	0.3618	

Significance codes: 0.0001 '***' 0.001 '**' 0.01 '*' 0.05 '.' 0.1 ' ' 1

Supplementary Table S6. ANOVA of culture-dependent rhizobacterial alpha diversity as represented by the Shannon diversity index. This is a two-way type III ANOVA.

Sources of Variation	Degrees of Freedom	Sum of Squares	F statistic	Pr(>F)	Statistical Significance
Fertilizer	1	0.2479	3.042	0.109	
Fertilizer: Rhizocompartment	1	0.2367	3	0.116	
Rhizocompartment	1	0.0021	0.025	0.876	

Significance codes: 0.0001 '***' 0.001 '**' 0.01 '*' 0.05 '.' 0.1 ' ' 1

Supplementary Table S7. Contingency table to assess the difference in distribution of isolates that tested positive or negative for solubilization of key plant nutrients, isolated from wheat grown in soils with and without fertilizer addition.

	Functional Bioassay	No. of isolates				Chi-squared test
		Non-fertilized wheat		Fertilized wheat		
		+ve	-ve	+ve	-ve	
	**Solubilization**					
**Rhizosphere**	Nitrate (Casein)	269	107	146	230	81****
	Phosphate	68	308	3	373	66****
	Potassium	62	314	17	359	29****
	Iron	50	326	7	369	35****
	Zinc	17	359	6	370	5.4*
	Salt tolerance	182	194	158	218	3.1
**Total PGPR**		306	70	163	213	116****
	**Solubilization**					
**Rhizoplane**	Nitrate (Casein)	329	47	255	121	42****
	Phosphate	168	208	7	369	193****
	Potassium	177	199	24	352	159****
	Iron	200	176	8	368	245****
	Zinc	50	326	102	274	22****
	Salt tolerance	190	186	215	161	3.3
**Total PGPR**		339	37	279	97	33****

Significance codes: 0.0001 '***' 0.001 '**' 0.01 '*' 0.05 '.' 0.1 ' ' 1

Supplementary Table S8. Differentially abundant 16S rRNA gene ASVs which were found to be enriched in culture-independent rhizosphere and rhizoplane samples from non-fertilized wheat and fertilized wheat.
See Supplementary_Table_S8.xlsx

Supplementary Table S9. Differentially abundant 16S rRNA gene ASVs which were found to be enriched in culture-dependent rhizosphere and rhizoplane samples from non-fertilized wheat and fertilized wheat. ASVs identified in both rhizocompartments are indicated in bold. 22 ASVs not classified to phylum level are not shown.
See Supplementary_Table_S9.xlsx

Supplementary Data.

Supplementary Data S1. Summary table of all bacterial isolates from *Triticum aestivum* rhizosphere and rhizoplane.
See Supplementary_Data_S1.xlsx

1. References

1. Kavamura VN, Hayat R, Clark IM, Rossmann M, Mendes R, Hirsch PR, et al. Inorganic Nitrogen Application Affects Both Taxonomical and Predicted Functional Structure of Wheat Rhizosphere Bacterial Communities. Front Microbiol. 2018;9:1074.
